# Dengue disease outbreak definitions are implicitly variable

**DOI:** 10.1016/j.epidem.2015.03.002

**Published:** 2015-06

**Authors:** Oliver J. Brady, David L. Smith, Thomas W. Scott, Simon I. Hay

**Affiliations:** a Spatial Ecology and Epidemiology Group, Tinbergen Building, Department of Zoology, University of Oxford, South Parks Road, Oxford, UK; b Fogarty International Center, National Institutes of Health, Bethesda, MD, USA; c Sanaria Institute for Global Health and Tropical Medicine, Rockville, MD, USA; d Department of Entomology and Nematology, University of California, Davis, CA, USA; e The Wellcome Trust Centre for Human Genetics, Roosevelt Drive, Oxford OX3 7BN, UK

**Keywords:** Outbreak, Response, Dengue, Decision-making, Policy

## Abstract

Infectious diseases rarely exhibit simple dynamics. Outbreaks (defined as excess cases beyond response capabilities) have the potential to cause a disproportionately high burden due to overwhelming health care systems. The recommendations of international policy guidelines and research agendas are based on a perceived standardised definition of an outbreak characterised by a prolonged, high-caseload, extra-seasonal surge. In this analysis we apply multiple candidate outbreak definitions to reported dengue case data from Brazil to test this assumption. The methods identify highly heterogeneous outbreak characteristics in terms of frequency, duration and case burden. All definitions identify outbreaks with characteristics that vary over time and space. Further, definitions differ in their timeliness of outbreak onset, and thus may be more or less suitable for early intervention. This raises concerns about the application of current outbreak guidelines for early warning/identification systems. It is clear that quantitatively defining the characteristics of an outbreak is an essential prerequisite for effective reactive response. More work is needed so that definitions of disease outbreaks can take into account the baseline capacities of treatment, surveillance and control. This is essential if outbreak guidelines are to be effective and generalisable across a range of epidemiologically different settings.

## Highlights

• With appropriate and timely control, disease outbreak burden can be minimised.
• Many different case data-based statistical methods are used to trigger outbreak response.
• Here we show that these methods are inconsistent and incomparable.
• This may hinder the effectiveness of outbreak response.
• Clear quantitative definitions of an outbreak are a prerequisite for effective outbreak early warning and response.

## 1 Introduction

While much progress has been made in our ability to treat and reduce the long-term burden of many infectious diseases (Lim et al., 2013), unexpected surges in case numbers above the seasonally expected mean can frequently derail progress or push already stretched healthcare resources to breaking point (Cotter et al., 2013; Garg et al., 2008; Hay et al., 2003a, 2003b). Disease outbreaks often develop rapidly, are difficult or impossible to predict and cause a disproportionately high burden due to the lack of response capabilities (Garg et al., 2008; Grais et al., 2007; Najera, 1999; WHO Ebola Response Team, 2014).

As a result of the clear importance of disease outbreaks to wider control efforts, research agendas and subsequent policy guidelines have heavily focussed on methods to predict outbreaks (early warning), how to identify them once they are occurring (early detection), how to respond to them appropriately (outbreak response protocols) and how to better plan for future outbreak occurrences (effective healthcare, surveillance and control resource allocation) (WHO, 2009, 2005, 1999; Farrar et al., 2007; Myers et al., 2000; Hutwagner et al., 2003). Optimisation of each of these individual goals is dependent on an unambiguous quantitative definition of exactly what the term “outbreak” refers to in terms of frequency, duration, amplitude and burden.

One common method for defining outbreaks is to use epidemiological criteria, where any temporal anomaly from the expected number of cases is classified as an outbreak (Wagner et al., 2001; Stroup et al., 1993). Distinguishing the expected number of cases (seasonal variation in incidence) from excessive case numbers (outbreaks) can be difficult for many communicable diseases that exhibit complicated transmission dynamics that are imperfectly sampled by health systems. Dengue, for example, is composed of four serotypes with complex patterns of cross-immunity in humans (Simmons et al., 2012; Wearing and Rohani, 2006) that are further complicated by highly heterogeneous environmentally-driven variations in each serotype's distribution and force of infection (Messina et al., 2014; Reiner et al., 2014). In addition to this, treatment-seeking, diagnosis and reporting of dengue is highly variable (Simmons et al., 2012; Endy et al., 2011; Brady et al., 2014), making interpretation of the seasonal signals in reported case data difficult (Hay et al., 2013).

Despite the complex heterogeneities in transmission of dengue virus (DENV) and many other infectious diseases, methods to distinguish baseline transmission from outbreaks remain simple, a feature that is often attributed to the capacity of health systems personnel to implement them. Many methods restrict their analysis to intra-annual trends by calculating a monthly or seasonal mean to define the baseline, often referred to as an endemic channel (Cullen et al., 1984). Outbreak thresholds are then often arbitrarily set at two standard deviations in excess of the endemic channel (Wagner et al., 2001; Stroup et al., 1993; WHO, 2009; Hutwagner et al., 2003). For many diseases, the remaining variation not accounted for by these methods means that (i) determining the endemic channel is highly uncertain and (ii) that the outbreak threshold line is exceeded frequently, briefly and sporadically (Badurdeen et al., 2013). These brief outbreaks may lead to considerable outbreak response measures being deployed, despite minimal excess cases. In public health or operational terms, such an occurrence would be considered as a false alarm for an outbreak.

The inappropriateness of some outbreak response plans to the original case data-based outbreak definitions for dengue has led many to adapt the endemic channel plus two standard deviations method to increase or decrease sensitivity and specificity, for example by requiring two successive weeks above the threshold before response activity is triggered (Harrington et al., 2013). This has led to many different dengue outbreak definitions being employed in different countries and regions (Harrington et al., 2013; Badurdeen et al., 2013). These individual definitions are at odds with international efforts to produce standardised, evidence-based outbreak response strategies (WHO, 2009; Pilger et al., 2010; Farrar et al., 2007; Hutwagner et al., 2003). International efforts are focusses on optimising responses to an idealised extra-seasonal surge-type outbreak that may or may not be relevant to the types of outbreaks identified by these locally adapted definitions.

**Fig. 1:**
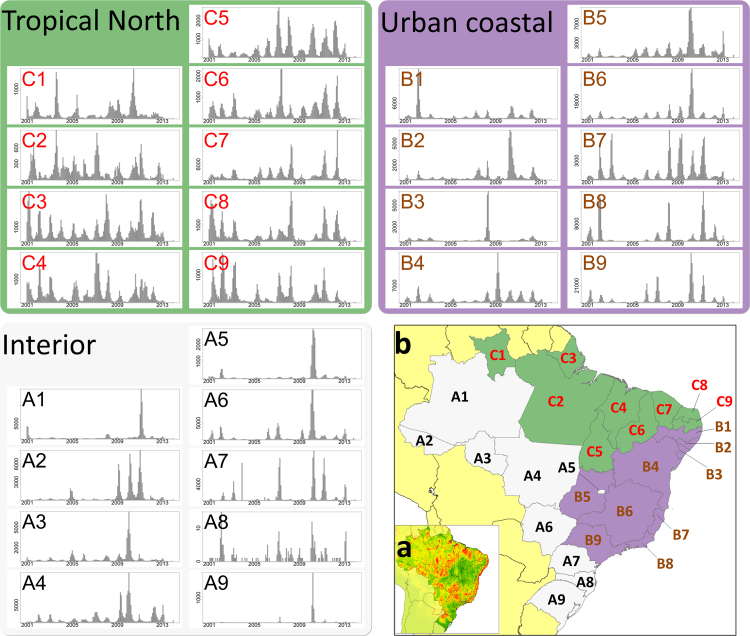
*Reported dengue case data in Brazil.* Each bar chart displays monthly reported dengue cases (suspected and confirmed) at a state level (*n* = 27) between the start of 2001 and the end of 2013. Map (a) shows the long-term average probability of dengue occurrence as determined by Bhatt et al. (2013). Map (b) shows the division of the 27 states into epidemiologically defined groups based on the epidemiological characteristics of their time series. States are divided as follows: Amazonas (A1), Acre (A2), Rondônia (A3), Matto Grosso (A4), Districto Federal (A5), Matto Grosso do Sul (A6), Santa Caterina (A7), Paraná (A8), Rio Grande do Sul (A9), Pernambuco (B1), Alagoas (B2), Sergipe (B3), Bahia (B4), Goiás (B5), Minas Gerais (B6), Espírito Santo (B7), Rio de Janeiro (B8), São Paulo (B9), Roraima (C1), Pará (C2), Amapá (C3), Maranhão (C4), Tocantins (C5), Piauí (C6), Ceará (C7), Rio Grande do Norte (C8), Paraíba (C9).

In this paper we use a dataset of reported dengue cases from Brazil (Fig. 1) to test a wide range of 102 existing outbreak definitions based on five endemic channels and their various parameterisations. This allows us to assess their comparability, consistency over a range of DENV transmission settings and timeliness in outbreak detection.

## 2 Methods

### 2.1 Dengue case data

We chose dengue in Brazil as a case study for comparing outbreak definitions for a number of reasons. First, Brazil has one of the most comprehensive dengue surveillance systems of any country, with over 200 million people under observation and data disaggregated monthly over ten years or more across 5570 municipalities. This data is also readily and freely available over long time periods from the Brazilian Ministry of Health surveillance system SINAN (Ministério da Saúde, 2014a, 2014b). Second, Brazil is also epidemiologically representative of a wide range of DENV transmission settings: In the tropical northern regions, year-round transmission enables relatively consistent transmission levels with some seasonal patterns (Fig. 1); in the densely populated cities of the southeast much of the case burden is concentrated in outbreaks that occur only once every few years and in the interior western regions, dynamics are dominated by rare large outbreaks that may only occur once every 10 years. Third, Brazil already dedicates significant resources towards vector control, including dengue, with over one billion USD being spent in 2008 (WHO, 2012), as well as, having an active research community interested in optimising how this money is spent. Brazil, therefore, is one of the most suitable countries to test and evaluate the usefulness of current dengue outbreak policy with a view to informing international policy guidelines on dengue outbreaks.

Monthly total dengue cases were extracted from January 2001 to December 2013 for 27 states (admin1 level (Food and Agriculture Organization of the United Nations, 2008)) from the Brazilian Ministry of Health surveillance system SINAN (Ministério da Saúde, 2014a, 2014b). Total dengue cases comprised of hospital reported, suspected and confirmed cases of dengue fever, dengue haemorrhagic fever and dengue shock syndrome. We chose to aggregate the municipality-level data to state-level as, in endemic settings, a more reliable seasonal signal can be obtained over this scale, meaning it is the more frequent scale at which outbreak identification for strategic planning occurs (Harrington et al., 2013). The robustness of our results to our choice of spatial and temporal scale is examined in the Supplementary information with evaluation at the municipality level, in the state of São Paulo, and using simulated weekly dengue cases at the national level.

### 2.2 Outbreak definitions

Existing outbreak definitions are composed of two components, (i) an endemic channel which aims to replicate a historical trend of expected cases and (ii) a set of criteria that determines what level of variation above this endemic channel is classified as an outbreak. In this analysis we aim to test the comparability of all endemic channels and their various parameterisations that make up the variety of outbreak definitions that are currently in use (Badurdeen et al., 2013).

There are five main methods used to calculate an endemic channel: recent mean, monthly mean, moving mean, cumulative mean and fixed incidence threshold, the calculation of which is summarised in Table 1 (Wagner et al., 2001; Stroup et al., 1993; Cullen et al., 1984). These methods are included in core policy documents published by the World Health Organization (WHO) (WHO, 2009) and Centers for Disease Control (CDC) (Hutwagner et al., 2003) and are widely used across a range of different diseases at different levels of transmission intensity (Hay et al., 2002; Badurdeen et al., 2013; Harrington et al., 2013). Calculation of each definition and its typical uses are given in Table 1. For our analysis, the fixed incidence threshold was set at 100 or 300 cases per 100,000 individuals (Lowe et al., 2014; Rigau-Perez et al., 1999; Badurdeen et al., 2013) with population data for each state obtained from the Instituto Brasileiro de Geografia e Estatística (IBGE) (IBGE, 2010). For all other methods the following variations on parameters used to define the base dataset for endemic channel calculation were explored: (i) the number of historical years to include: five (current) or all available (long-term); and (ii) the inclusion of outbreak years in the base dataset, yes or no. Outbreak years within the base dataset were defined by the total annual number of cases exceeding two standard deviations of any combination of three or more historical years. This is a practical approach to “trimming”, an approach that minimises the effect of long-tailed distributions on the historical mean and is often implemented by excluding arbitrarily or quasi-quantitatively determined “outbreak years” to increase sensitivity of the contemporary outbreak definition. While “outbreak years” and “outbreaks” do not necessarily have to overlap, there may be some circularity in defining outbreaks using a dataset that has already had outbreak years arbitrarily defined and removed.

The following parameters that determine the level of variation above the endemic channel that defines an outbreak were also explored: (i) the number of standard deviations above the historical mean: 1 or 2 and (ii) the number of monthly observations above the threshold that would trigger the start of an outbreak: 1, 2 or 3. The four dynamic endemic channel methods, combined with the four base dataset parameters and the six outbreak threshold parameters gave a total of 96 (=4 × 2 × 2 × 2 × 3) definitions which combined with the six definition variations of the fixed incidence method (two thresholds that just vary by the number of observations above the threshold to trigger an outbreak), gave a total of 102 different outbreak definitions.

Each outbreak definition was calculated annually at the beginning of years 2006–2013, then its performance was evaluated over the following year, with the exception of the recent mean method which was evaluated on a rolling monthly basis. The following measures were collected: the number of outbreaks identified and the proportion of total cases in the time series that exceeded the outbreak threshold.

To quantify the relative variation in outbreak characteristics identified by each definition, a kernel estimation method was used. The two variables, number of outbreaks identified and the proportion of total cases in the time series that exceeded the outbreak threshold, were first scaled to equivalent maximum ranges (0–10). Using the *hdrcde* package in R, a two dimensional highest density region method was used to estimate the area of the kernel that contained the most concentrated 50% of the density of the combined distribution (Hyndman, 1996). This allowed us to measure relative variation in outbreak characteristics, between for example two different states, by taking into account the spread of the highest density regions. A dispersal index, *D*, quantified this relative dispersal with higher values indicating more varied outbreak characteristics.

## 3 Results

### 3.1 Different outbreak definitions are incomparable even when applied to the same data

**Fig. 2:**
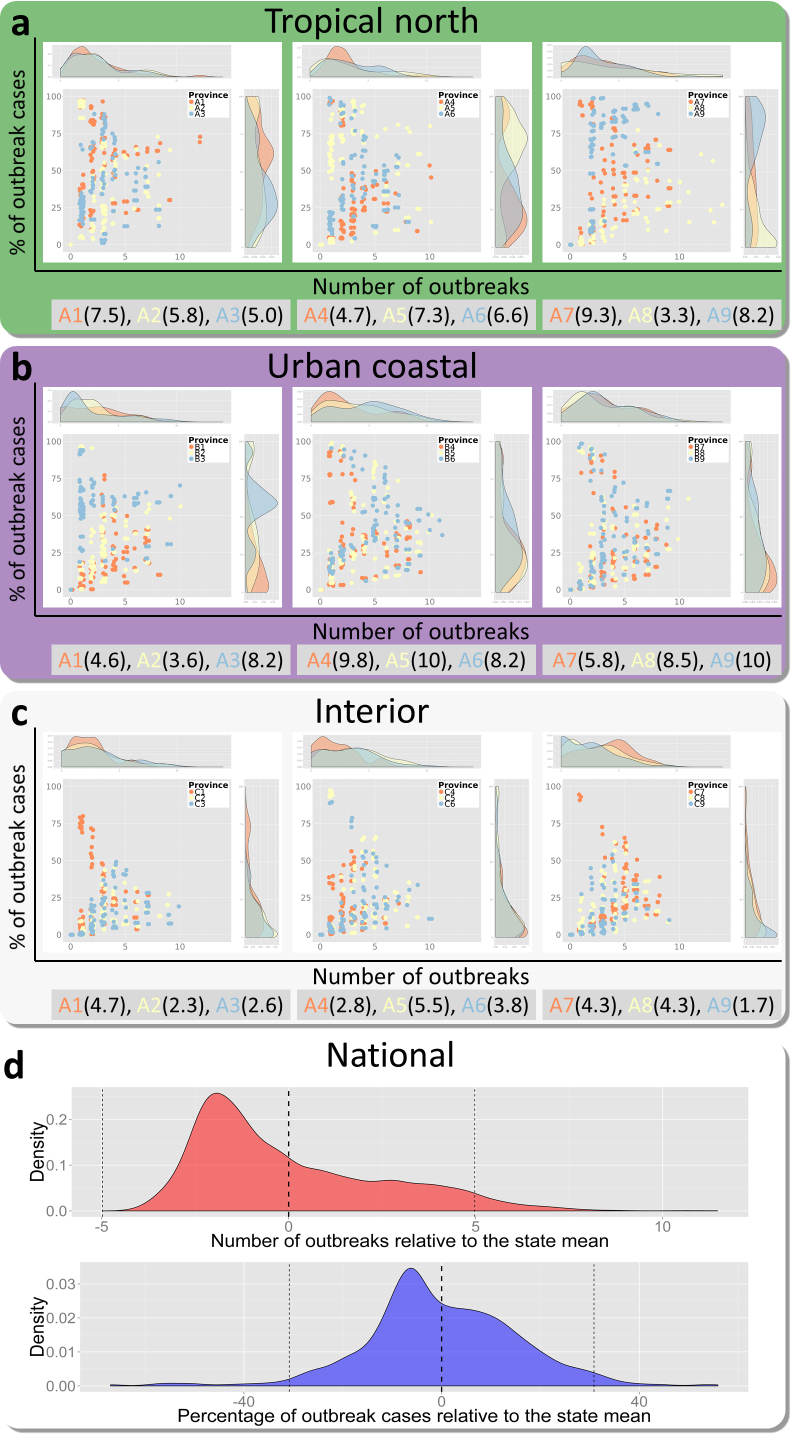
*Variability between all outbreak definitions applied to grouped states.* Parts (a–c) show the distribution of the number of outbreaks and percentage of outbreak characteristics identified (the two axes) when each definition (102 same coloured points) is applied to the same state (different colours, grouped three states per plot). This variance around the mean of each state is aggregated to give the distribution at the national level in d. Dotted black lines in d show the mean and 95% confidence intervals.

To compare the outbreak characteristics identified by each different outbreak definition we applied every definition to each state in Brazil. To compare outbreak characteristics we measured the total number of unique outbreaks identified by each definition and the percentage of total cases across the time series that were classified as outbreak cases. The distribution of these results for each state is shown grouped by the three epidemiological categories in Fig. 2a–c and the aggregated results showing variation around the mean of each state is shown in Fig. 2d.

Overall outbreak characteristics are highly variable (Fig. 2d). Among the majority of definitions (95%), the total number of outbreaks identified by any given pair of definitions could differ by as many as 10 outbreaks and the total proportion of outbreak cases could differ by as much as 65% over just a nine-year time series. This variability is highest in the urban coastal regions (average dispersion metric $\hat{D}=7.6$), still high in the tropical north regions ($\hat{D}=6.4$), and lowest in the interior region ($\hat{D}=3.6$) (Fig. 2). For the interior states variability mainly occurs in the number of outbreaks identified (Fig. 2c) due to definition sensitivity in already low transmission settings, while variation in the tropical north mainly occurs in the proportion of cases identified as outbreaks (Fig. 2a) due to the similarities between seasonal trends and the small outbreaks they experience (Fig. 1). In the urban coastal region we see high variation in both of these axes (Fig. 2b) highlighting the difficulty in distinguishing frequent large outbreaks from seasonal trend, such as in Espírito Santo (B7, Fig. 1)

### 3.2 Even if outbreak definitions are standardised, outbreak characteristics are inconsistent in different states

**Fig. 3:**
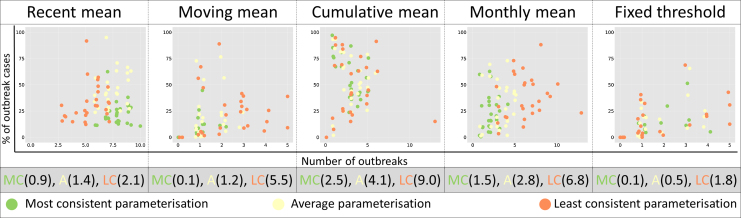
*Variability of outbreak characteristics when one outbreak definition is applied to all 27 states.* The scatterplots show the outbreak characteristics of each state (shown by 27 same coloured dots) when the most consistent (green), least consistent (orange) and an average (yellow) defintion is chosen from each endemic channel.

While outbreak characteristics are clearly variable between different outbreak definitions, thus hampering inter-definition comparability, in many cases a particular single definition will be chosen and applied over a wide number of areas, such as a nationally standardised definition. To test the consistency and comparability of such an approach we measured variation in outbreak characteristics when applying a single definition to all 27 Brazilian states (Fig. 3).

No one single definition identified consistent outbreak characteristics when applied nationally (Fig. 3). While some outbreak definitions clearly identified outbreaks with more variable characteristics than others, the general trend was that even the most consistent outbreak definition offered little improvement in consistency over the average definition and there were no common themes among the parameters that made definitions more consistent (Table 2). Where outbreak definitions did reach consistency, such as the most consistent moving mean definition (Fig. 3), this was often due to identifying no outbreaks, or outbreaks of limited duration. As the choice of outbreak definition parameterisation is often justified by improved geographic relevance, this result suggests little improvement in consistency is gained by minor changes in definition parameterisation at the national level.

**Fig. 4:**
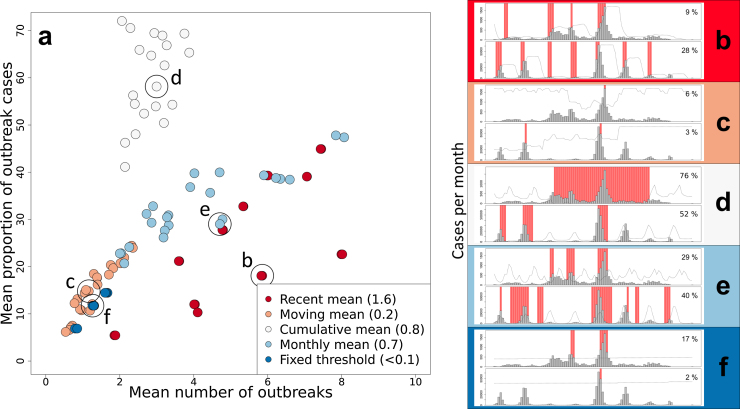
*Outbreak characteristic variability by endemic channel parameterisation.* (a) Shows the mean outbreak characteristics across all states of each endemic channel parameterisation (*n* = 24 except fixed threshold where *n* = 6 unique coloured dots). Representative examples of each of the endemic channel definition types applied to low transmission (Roraima, C1, upper panel) and high transmission (São Paulo, B9, lower panel) environments are shown in (b–f). Grey bars indicate monthly case numbers 2006–2013, dotted lines show the endemic channel for each year and red background indicates outbreak months identified by the given definition. The percentage figure in the top right shows the percentage of total cases that are identified as outbreak cases.

Outbreak definitions based on cumulative mean and monthly mean endemic channels tended to identify a greater proportion of total cases as outbreaks, while monthly mean and recent mean methods tended to identify the highest number of outbreaks (Fig. 4). Fixed mean and moving mean endemic channels tended to identify outbreaks of smaller magnitude, but also fewer outbreaks (Fig. 4). Recent mean, cumulative mean, and monthly mean-based definitions appeared to be the most flexible and could be tweaked to identify outbreaks of different magnitudes and frequencies $\left(\hat{D}=1.6,\;0.8\;\text{and}\;0.7,\;\text{respectively}\right)$. Representative examples from each endemic channel type are explored in more detail in Fig. 4b–f where each definition is applied to a low incidence (Roraima, C1, upper panel) and high incidence (São Paulo, B9, lower panel) setting. The types of outbreaks identified in these two contrasting DENV transmission settings vary considerably in terms of (i) the characteristics of each outbreak (how many cases and over how long) and (ii) outbreak frequency and intra-annual timing. This suggests that even if a standardised definition of an outbreak was adopted, the actual characteristics of an outbreak, and thus approaches and resources needed to respond, would be significantly different in different states.

Finally, it is also worth noting that even when one definition is applied consistently in one state, the characteristics of the outbreaks identified across time are inconsistent (Fig. 4b–f). The outbreaks identified vary in size, length and in the times of year in which they occur.

### 3.3 The timing of onset of an outbreak varies significantly depending on outbreak definition

**Fig. 5:**
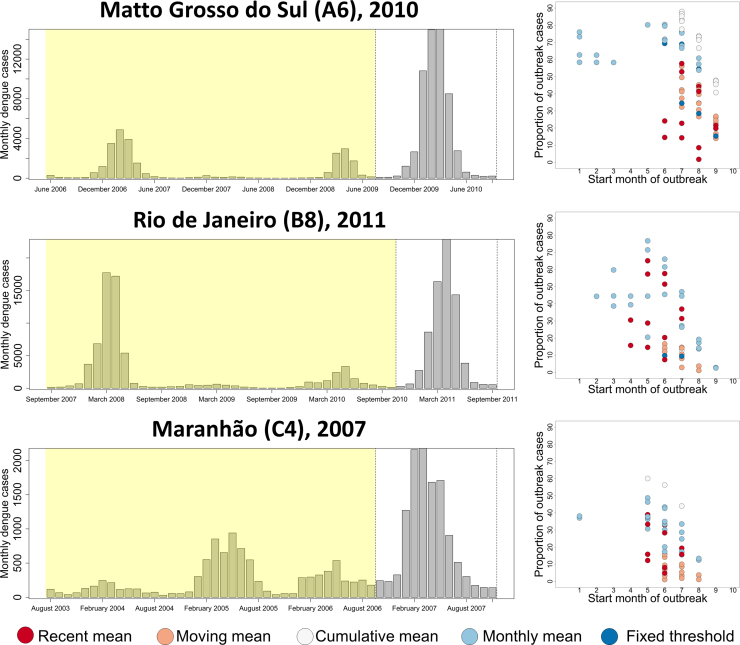
The difference in time of onset and overall outbreak size for different outbreak definitions applied to different example outbreaks. The longitudinal plot (left) shows the monthly reported case number in the four years (shaded yellow) building up to an extra-seasonal surge in cases (unshaded) in three different states with differing DENV transmission dynamics. For each outbreak, the graph on the right shows the variability in timeliness of detection (number of months since outbreak onset, *x*-axis) and outbreak size as a proportion of total cases (*y*-axis) when fitted to data in the yellow shaded region and applied to the unshaded region. Only definitions that identified an outbreak are shown.

For the purpose of early detection and rapid response to disease outbreaks, a useful definition needs to identify the onset of an outbreak, and trigger appropriate response activities before the bulk of cases occur. The earlier response measures are enacted during the onset of an outbreak, the greater the chance interventions will have at reducing the number of excess cases. To test the variation in sensitivity of timeliness of outbreak detection, we analysed the predictions of all 102 definition combinations against outbreaks in an interior state (Matto Grosso do Sul, A6, 2010), an urban south eastern state (Rio de Janeiro, B8, 2011) and a northern state (Maranhão, C4, 2007) all with differing DENV transmission dynamics (Fig. 5).

When focussing on a single outbreak there is considerable variation in timeliness of detection and resultant outbreak size between different outbreak definitions. In all three states different definitions identified outbreaks that varied in their timing of outbreak onset by as much as seven or eight months, with the majority of outbreaks still varying by as much as four months (Fig. 5). Generally, monthly mean and recent mean methods proved the timeliest, while moving mean methods were consistently the least timely of all methods tested. Over all three outbreaks it can be observed that even definitions that lag behind those that identify outbreaks early, can still identify the bulk of cases in a given outbreak. If outbreak response is primarily reactive, these less sensitive definitions would be more suitable, however if outbreak response is preventative, the additional lead time afforded by more sensitive definitions may be of benefit. As a final point, it should be noted that at least the majority of definitions did identify an outbreak in these three selected examples and while the timing and scale of responses would likely be very different depending on which outbreak definition was used, at least a response would have been triggered.

## 4 Conclusions and discussion

While the concept of a disease outbreak is undoubtedly important, it is clear there is no consensus on how to define such a term. Moreover, the range of existing definitions is incomparable, inconsistent and highly variable in their ability to permit effective early response. The magnitude of this incomparability also increases with outbreak size, and thus disease burden. These findings present a real concern for existing and planned policy guidelines, as well as operational early warning and early detection systems, that have given insufficient consideration to the heterogeneous outbreak characteristics identified in real time in a variety of different DENV transmission settings (WHO, 2009, 2005, 1999; Farrar et al., 2007; Myers et al., 2000; Hutwagner et al., 2003; Harrington et al., 2013).

In this analysis we have shown that even if a single outbreak definition was adopted from among the range of different definitions currently available, exactly what constitutes an outbreak would still be inconsistent both across space and through time. This has implications for national level outbreak response guidelines as it does not present a standardised measure with which to compare sub-national healthcare and control needs, nor does it allow the monitoring of progress towards long-term reductions in disease burden. Furthermore, even using a standardised definition within a single state to optimise outbreak response strategies may be inadequate as the characteristics of outbreaks may change over time despite only subtle changes in DENV transmission dynamics.

As an additional limitation, we showed that currently-used outbreak definitions vary widely in their capacity to enable effective preventative outbreak measures such as early detection and early response (Lowe et al., 2014; Chowell et al., 2008). This may make the recommendation of such activities obsolete under certain conditions with particular outbreak definitions, particularly those with reduced sensitivity that require more than one observation above a given threshold to trigger an outbreak. In such a situation, preventative control may be more heavily reliant on early warning systems that predict outbreaks based on temporal anomalies in epidemiological and environmental warning signals (Lowe et al., 2014; Degallier et al., 2010; Teklehaimanot et al., 2004a, 2004c). Detecting these temporal anomalies in different kinds of data may well encounter similar issues with inconsistency and variability that have been highlighted here. We would therefore recommend the use of techniques that are more complex than simple interpretations of historical means or fixed thresholds to detect these temporal anomalies. Particular care must be taken in making arbitrary decisions about the data used to define the historical baseline, such as excluding “outbreak years”. Even with the use of more flexible statistical frameworks (Box et al., 2013) and a wealth of new data (Ministério da Saúde, 2014a, 2014b; PAHO, 2014), the identified associations may well be highly dependent on how disease outbreaks are defined. A clear definition of a disease outbreak is therefore an essential prerequisite for the evaluation of the sensitivity and specificity of forecasting, early warning, or early detection. Without a meaningful and operationally relevant fixed definition, instead of just arbitrary thresholds (Lowe et al., 2014, 2013), there is no way to compare the performance of predictive models, nor is it possible to construct guidelines based on their outputs. These criteria need to be rigorously assessed before any predictive or early warning system can become a practical tool for outbreak identification and response.

The recommendations presented here come from the analysis of reported, suspected and confirmed dengue cases in Brazil. This was done to maximise the available data and to avoid the effects of discrepancies in laboratory diagnostic capacities between different states. While spatial and temporal variation in misreporting are likely to affect the ability of outbreak definitions to identify consistent outbreaks, it is likely that the common propensity for over-reporting of dengue cases during outbreaks and underreporting at other times would only reinforce the consistent distinction between outbreak periods and non-outbreak periods (Teutsch and Churchill, 2000). It is possible, however, that mis-reporting could affect the timeliness of outbreak detection, which was not tested here.

We chose a subset of the wide variety of outbreak definitions available (Debin et al., 2013; Pelecanos et al., 2010; Jafarpour et al., 2013; Dórea et al., 2013; Polanco et al., 2013; Chen and Chang, 2013; Hay et al., 2003a). Our selection was based upon the principle types of definitions recommended in current outbreak guidelines (WHO, 2009; Hutwagner et al., 2003), with proven uptake in selected countries (Table 1) and some claims of consistent results (Zhang et al., 2014). Additional definitions may identify more consistent outbreak characteristics, but their performance over wide-scale datasets have yet to be tested. We chose to evaluate outbreak definitions at the state scale as it is the most common level for strategic wide-scale outbreak response recommendations to be made (Harrington et al., 2013). Given the general increase in heterogeneity at lower spatial scales and over shorter time periods, we would not expect existing outbreak definitions to be any more comparable at lower spatial scales or with more frequent reporting intervals. This is supported by the results of our further analysis presented in the Supplementary information. It is possible that outbreak definitions in transmission-free settings (where any occurrence of cases typically triggers outbreak response (Harrington et al., 2013)) may be consistent and appropriate; this definition has not been tested in this analysis. Despite this, given the diverse nature of global DENV transmission (Bhatt et al., 2013; Messina et al., 2014), of which Brazil represents a small subset, we would not expect outbreak definitions to be any more comparable in different countries or regions. We also confine our analysis of disease outbreaks to dengue. While the four serotypes of dengue and their associated immune responses in humans mean patterns of DENV transmission are highly spatially and temporally heterogeneous, various intricacies of transmission in many other diseases (Reiner et al., 2013; Smith et al., 2014; Anderson and May, 1991) are likely to lead to similar complex reported case dynamics and equally unclear definitions of what comprises a disease outbreak. Similar analyses for other diseases should be conducted to test this hypothesis and to evaluate the usefulness of more established international guidelines for outbreak response.

For dengue, and many other diseases, information other than reported and suspected cases is available which may be more reliable for defining outbreaks due to the inherent temporal biases of reporting and diagnosis associated with simple reported case data (Klaucke, 1994). Data on the percentage of positive diagnostic tests, entomological indices and environmental signals (Harrington et al., 2013; Teklehaimanot et al., 2004b) can be incorporated alongside reported cases in defining disease outbreaks or pre-emptive alert phases. Following an alert phase, an outbreak investigation is typically triggered which may involve forms of sentinel or enhanced passive surveillance. Despite these recommendations, what defines an outbreak following the results of these investigations remains unclear in many cases (Harrington et al., 2013). Irrespective of their reliability, data additions are often logistically and financially costly to make timely and outbreak-relevant, making them counter-productive to the goal of internationally standardised outbreak response and less useful in resource-constrained settings. Furthermore, this does not represent outbreak identification in the majority of settings for dengue (Harrington et al., 2013; Badurdeen et al., 2013). It is also likely that many of these additional data types are closely correlated with reported dengue cases, in which case the benefit of improved outbreak detection consistency needs to be weighed up against the cost of collecting the additional data. If such additional data sources are found to be useful in defining a dengue outbreak, it should be emphasised that they should only be considered in addition to, and not at the expense of, routine passive surveillance of suspected and confirmed dengue cases. This basic measure has core value in identifying dengue seasonality (Hay et al., 2013) and detecting dengue outbreaks as it is the most direct measure of the number of people placing a burden on the healthcare system, in addition to being the most abundant data source for pattern recognition.

Given that dengue and likely many other diseases have frequent, varied and unpredictable deviations from seasonal mean case numbers, there is a rationale to reconsider what the terms “disease outbreak” and “outbreak response” refer to. In epidemiological terms, outbreaks occur at many different levels of intensity, duration and velocity in deviations from seasonal means, driven by a range of factors that determine transmission suitability (Brady et al., 2013; Wearing and Rohani, 2006; Johansson et al., 2009). From a public health perspective, however, the term “outbreak” refers to a situation where routine surveillance, treatment and control capacities are exceeded and exceptional interventions are required. When we use epidemiological data to differentiate routine from excessive caseloads we assume that routinely deployed public health resources can be, and are, matched to the identified baseline transmission levels. For a number of logistical, financial and practical reasons this is unlikely to be the case, especially with some of the more variable outbreak definitions. Furthermore, many of these resources, such as patient beds in a hospital, are required for many different diseases and conditions and therefore have varying degrees of flexibility.

If the purpose of an outbreak definition is to identify periods of excess burden, a thorough assessment of baseline healthcare surveillance, control, and treatment capacities is needed. Measures such as the number of hospital beds, insecticide stockpiles and the quantity of surveillance teams will all be important in defining the baseline capacity limits. Additional data on actions that are taken in outbreak times, such as staff surge capacities, resources for additional mass fogging, rapid sentinel surveillance and the speed of deployment of each of these, will then be useful for defining what numbers of cases per month should fall under the remit of routine activities and what number of cases should trigger exceptional measures. Given this data, modelling approaches can be used to optimally allocate resources between routine and exceptional responses given the logistical constraints of each resource. Such a model could be evaluated periodically and have a role in healthcare budget allocation as well as providing recommendations for specific actions to be undertaken in outbreak times. Any integration of early warning or early detection systems needs to be critically evaluated against investments in improvements of resource allocation or improved disease surveillance.

This alternative method of defining an outbreak may require unconventional and additional data sources, but has the potential to add much-needed clarity to the neglected issue of what is a practical definition of an outbreak. Only then is it feasible to undertake quantitative modelling approaches that aim to optimise prevention or amelioration activities that minimise the burden of dengue outbreaks. The scale and extent of Brazil's dengue surveillance system means it is well-placed to advance dengue outbreak research and could be a first adopter of a new generation of evidence-based dengue outbreak policy.

## Author contributions

OJB, DLS, TWS and SIH designed the experiment. OJB, wrote the manuscript and collected and analysed the data. All authors helped with data and results interpretation and were involved in drafting, revising and final approval of the manuscript.

## Competing financial interests

The authors declared no financial interests.

## Figures and Tables

**Table 1:** *Endemic channel definitions.* Equations calculate the mean (*μ*), standard deviation (*σ*) and critical threshold (*T*_*c*_) for observations at time point *i* for each of the five methods. Selected methods can be modified by changing the number of years (*b*) in the baseline dataset, or by altering the number of standard deviations (*k*) that define the critical threshold.

	Recent mean (EARS C1 and C2)	Monthly mean (historical limits method)	Moving mean (smoothed mean)	Cumulative mean	Fixed incidence threshold
**Countries using method**	USA for respiratory illnesses (Hutwagner et al., 2003). Similar methods for dengue in Indonesia (Badurdeen et al., 2013)	Colombia, Dominican Republic, Peru and Vietnam for dengue (Badurdeen et al., 2013)	Brazil, Malaysia and China for dengue (Zhang et al., 2014; Badurdeen et al., 2013). USA for multiple diseases including dengue (Rigau-Perez et al., 1999)	USA for *Salmonella* (Hutwagner et al., 2003). Proposed for malaria in Thailand (Cullen et al., 1984). Ros River virus in Australia (Pelecanos et al., 2010)	Puerto Rico and Brazil for dengue (Rigau-Perez et al., 1999; Badurdeen et al., 2013; Lowe et al., 2014)
**Typical uses**	Diseases with little seasonal pattern and limited surveillance data	Diseases with a consistent seasonal cycle	Diseases with a seasonal cycle, the timing of which shifts year on year	Diseases with sporadic outbreaks	Diseases where response capacity is set to a particular fixed level of incidence
**Method of calculation**	The overall mean of a small set of recent observations (Hutwagner et al., 2003)	The mean of the corresponding months in the base dataset (Cullen et al., 1984)	The mean of the corresponding months and three months either side in the base dataset (Cullen et al., 1984)	The mean of the corresponding months yearly cumulative case count (Hutwagner et al., 2003)	A chosen fixed value of cases per 100,000 individuals in the population (Lowe et al., 2014)
**Equation**	$\mu_{i}=\frac{\sum_{b=s}^{b=t}\left(x_{\left(i-b\right)}\right)}{7}$ $\sigma_{i}=\sqrt{\frac{\sum_{b=s}^{b=t}{\left(x_{\left(i-b\right)}-\mu_{i}\right)}^{2}}{6}}$*T*_*c*,*i*_ = *μ*_*i*_ + *kσ*_*i*_ where the data window is defined by: *s* = 1 and *t* = 7 for C1 and *s* = 3 and *t* = 9 for C2	$\mu_{i}=\frac{\sum_{b=1}^{b=b}\left(x_{\left(i-12b\right)}\right)}{b}$ $\sigma_{i}=\sqrt{\frac{\sum_{b=1}^{b=b}{\left(x_{\left(i-12b\right)}-\mu_{i}\right)}^{2}}{\left(b-1\right)}}$*T*_*c*,*i*_ = *μ*_*i*_ + *kσ*_*i*_	$\begin{array}{c} \mu_{i}=\frac{\sum_{b=1}^{b=b}\left(x_{\left(i-12b-1\right)}+x_{\left(i-12b\right)}+x_{\left(i-12b+1\right)}\right)}{b}\\ \sigma_{i}=\sqrt{\frac{\sum_{b=1}^{b=b}{\left(x_{\left(i-12b-1\right)}-\mu_{i}\right)}^{2}+{\left(x_{\left(i-12b\right)}-\mu_{i}\right)}^{2}+{\left(x_{\left(i-12b+1\right)}-\mu_{i}\right)}^{2})}{\left(b-1\right)}}\\ T_{c,i}=\mu_{i}+k\sigma_{i} \end{array}$	$\mu_{i}=\frac{\sum_{b=1}^{b=12b}\left(x_{\left(i-b\right)}\right)}{12b}$ $\sigma_{i}=\sqrt{\frac{\sum_{b=1}^{b=b}{\left(x_{\left(i-12b\right)}-\mu_{i}\right)}^{2}}{\left(b-1\right)}}$*T*_*c*,*i*_ = max(0, [*σ*_*i*_((*k* + 0.5) − *T*_*c*,(*i*−1)_) + *μ*_*i*_])	*T*_*c,i*_ = 0.001 or 0.003

**Table 2:** *Parameter details of the most and least consistent definitions shown in*Fig. 3*.* Parameters are explained in the methods section. Fixed threshold methods use fixed values of incidence (100 or 300 cases per 10,000) instead of standard deviations above the mean.

Endemic channel	Parameterisation	Years of baseline data	Number of consecutive observations above threshold	Outbreak years included	Standard deviations above mean	Relative consistency (*D*)
Recent mean	Most cons.	5	1	No	1	0.9
	Average	All	1	Yes	1	1.4
	Least consistent	All	2	Yes	1	2.1

Moving mean	Most cons.	All	3	Yes	2	0.1
	Average	5	1	No	2	1.2
	Least consistent	All	1	No	1	5.5

Cumulative mean	Most cons.	5	3	Yes	1	2.5
	Average	All	3	Yes	1	4.1
	Least consistent	All	1	Yes	2	9.0

Monthly mean	Most cons.	5	3	No	2	1.5
	Average	All	2	No	2	2.8
	Least consistent	All	1	Yes	1	6.8
Fixed threshold	Most cons.	–	3	–	0.01 fixed	0.1
	Average	–	2	–	0.01 fixed	0.5
	Least consistent	–	1	–	0.01 fixed	1.8

cons. = consistent
