# Supplementary material for: Dengue disease outbreak definitions are implicitly variable
Source: Epidemics. 2015 Jun;11:92–102. doi: 10.1016/j.epidem.2015.03.002 (PMC4429239; doi:10.1016/j.epidem.2015.03.002)
Supplement: Supplementary file 1 [file mmc1.docx]

**Supplementary Information**

**This Supplementary Information includes:**

1. **Supplementary figures:**

- Supplementary figure S1. Variability between state and municipality level outbreak characteristics among all outbreak definitions
- Supplementary figure S2. Variability between state and municipality level outbreak characteristics when just one outbreak definition is applied
- Supplementary figure S3. Variability between outbreak characteristics among all outbreak definitions when applied to monthly or weekly case data
- Supplementary figure S4. Variability between outbreak characteristics when just one outbreak definition is applied to monthly or weekly case data
- Supplementary figure S5. The difference in time of onset and overall outbreak size for three example outbreaks when all definitions are applied to weekly or monthly case data

1. **Supplementary Results:**

- Comparison of outbreak characteristics when standard outbreak definitions are applied to municipality instead of state level dengue case data
- Comparison of outbreak characteristics when standard outbreak definitions are applied to weekly instead of monthly dengue case data

1. **Supplementary Methods:**

- Assembly of municipality level data for the state of São Paulo
- Simulation of comparable weekly data from monthly datasets

**Supplementary figures**

**
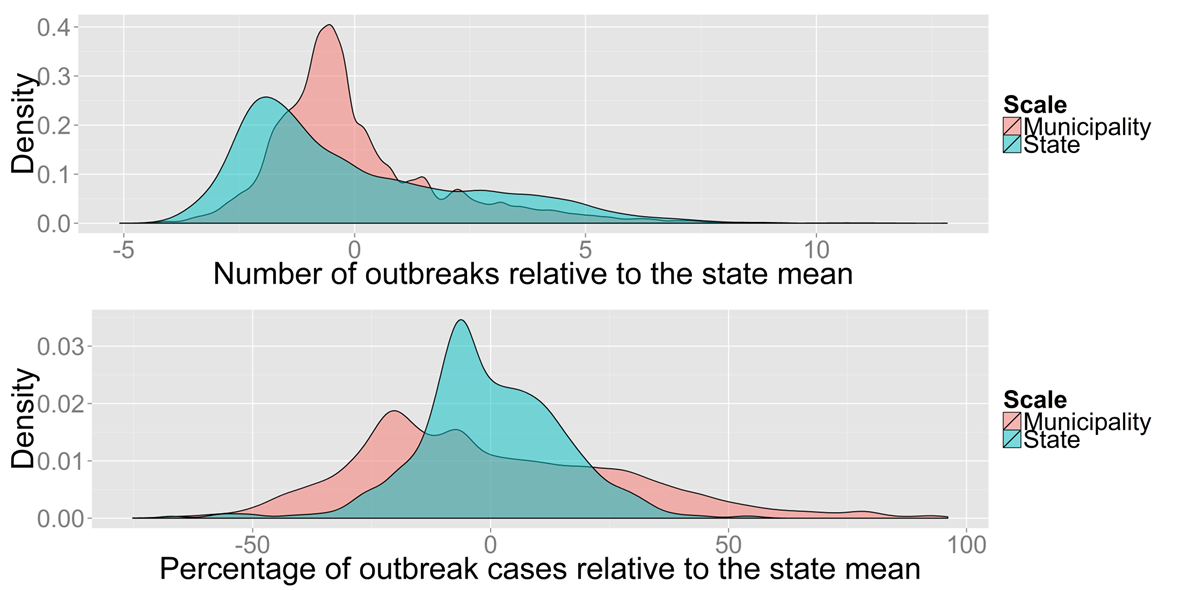
**

**Supplementary figure S1. Variability between state and municipality level outbreak characteristics among all outbreak definitions.** This figure summarises the variation in the number of outbreaks identified (upper panel) and the percentage of cases classified as an outbreak (lower panel) across all outbreak definition variations when applied to the 602 municipalities of São Paulo (red) or the 27 states of Brazil (blue). This figure is analogous to figure 2d in the main manuscript.

**
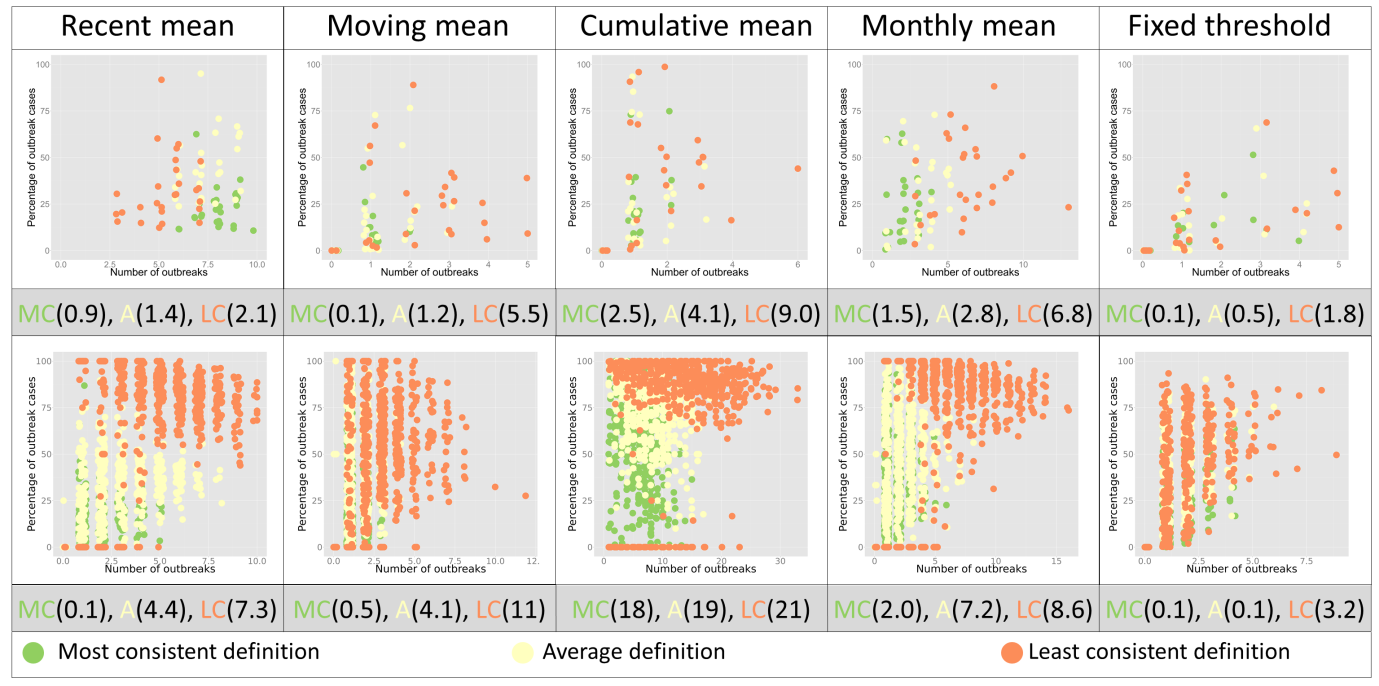
Supplementary figure S2. Variability between state and municipality level outbreak characteristics when just one outbreak definition is applied.** This figure shows the variation in outbreak characteristics when just a single definition is applied to the 27 states (upper panel) of Brazil or the 602 municipalities (lower panel) of São Paulo. The most consistent definition (equally weighted between the number of outbreaks and percentage of outbreak cases identified) across the different geographic regions is shown in green, while the average definition is shown in beige and the least consistent in orange. These are divided by different endemic channel types. This figure is analogous to figure 3 in the main manuscript.

| Endemic channel | Parameterisation | Years of baseline data | Number of consecutive observations above threshold | Outbreak years included | Standard deviations above mean | Relative consistency ($D$) |
| --- | --- | --- | --- | --- | --- | --- |
| Recent mean | Most consistent | 5 | 3 | No | 2 | 0.1 |
|  | Average | 5 | 2 | Yes | 1 | 4.4 |
|  | Least consistent | All | 1 | Yes | 1 | 7.3 |
| Moving mean | Most consistent | 5 | 3 | Yes | 2 | 0.5 |
|  | Average | All | 2 | No | 2 | 4.1 |
|  | Least consistent | All | 1 | Yes | 1 | 11 |
| Cumulative mean | Most consistent | 5 | 3 | Yes | 2 | 18 |
|  | Average | 5 | 2 | No | 2 | 19 |
|  | Least consistent | 5 | 1 | No | 1 | 21 |
| Monthly mean | Most consistent | 5 | 3 | Yes | 2 | 2.0 |
|  | Average | All | 2 | Yes | 2 | 7.2 |
|  | Least consistent | All | 1 | No | 1 | 8.6 |
| Fixed threshold | Most consistent | - | 3 | - | 0.01 fixed | 0.1 |
|  | Average | - | 2 | - | 0.01 fixed | 0.1 |
|  | Least consistent | - | 1 | - | 0.01 fixed | 3.2 |

**Supplementary table S1. Parameter details of the most and least consistent definitions shown in Supplementary figure S2.** Parameters are explained in the methods section of the main manuscript. Fixed threshold methods use fixed values of incidence (100 or 300 cases per 10,000) instead of standard deviations above the mean.


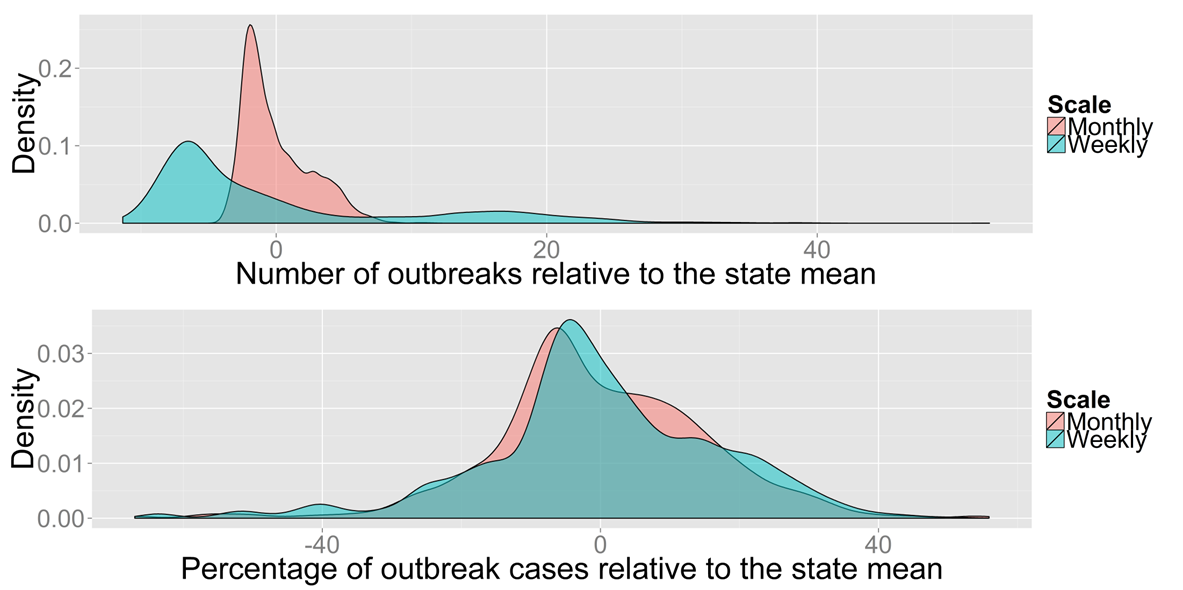


**Supplementary figure S3. Variability between outbreak characteristics among all outbreak definitions when applied to monthly or weekly case data.** This figure summarises the variation in the number of outbreaks identified (upper panel) and the percentage of cases classified as an outbreak (lower panel) across all outbreak definition variations when applied to the monthly dengue case data (red) or simulated weekly case data (blue). This figure is analogous to figure 2d in the main manuscript.


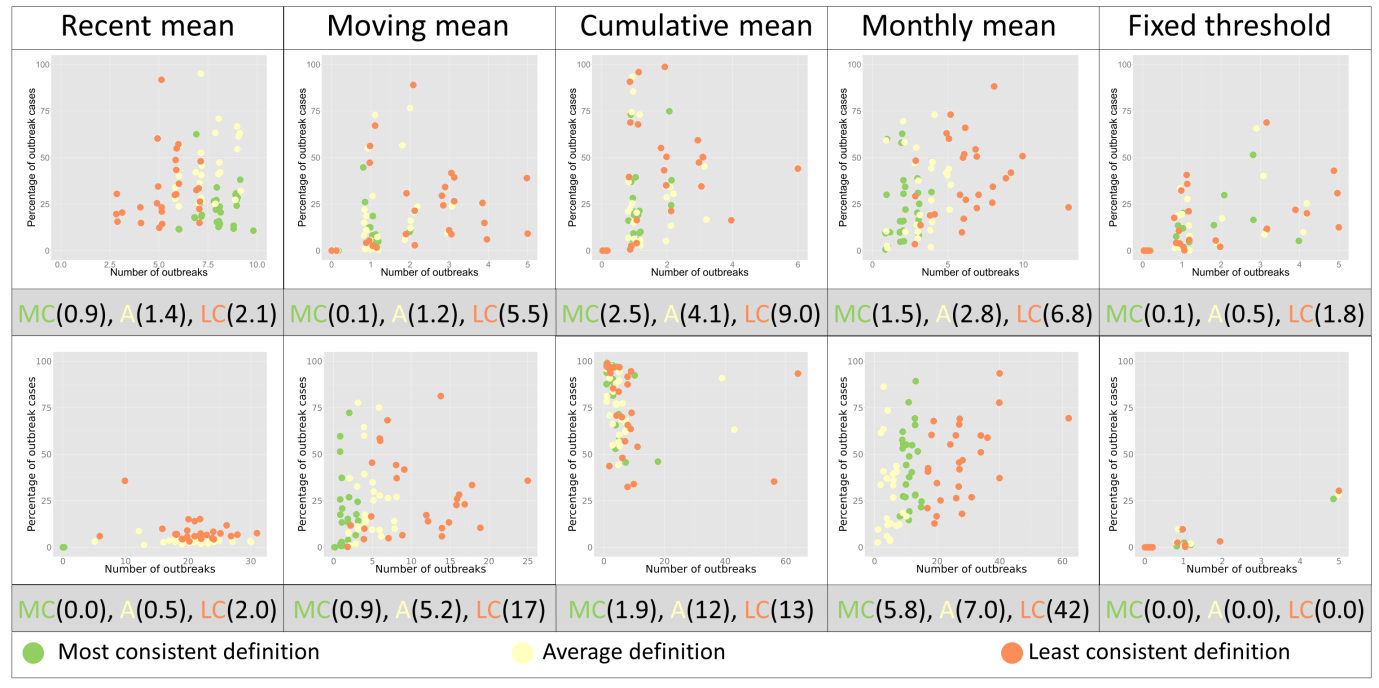


**Supplementary figure S4. Variability between outbreak characteristics when just one outbreak definition is applied to monthly or weekly case data.** This figure shows the variation in outbreak characteristics when just a single definition is applied to monthly dengue case data (upper panel) or simulated weekly case data (lower panel). The most consistent definition (in terms of both number of outbreaks and percentage of outbreak cases identified) across the different geographic regions is shown in green, while the average definition is shown in beige and the least consistent in orange. These are divided by different endemic channel types. This figure is analogous to figure 3 in the main manuscript.

| Endemic channel | Parameterisation | Years of baseline data | Number of consecutive observations above threshold | Outbreak years included | Standard deviations above mean | Relative consistency ($D$) |
| --- | --- | --- | --- | --- | --- | --- |
| Recent mean | Most consistent | 5 | 3 | No | 2 | 0.0 |
|  | Average | All | 3 | Yes | 2 | 0.5 |
|  | Least consistent | All | 3 | Yes | 1 | 2.0 |
| Moving mean | Most consistent | All | 3 | No | 2 | 0.9 |
|  | Average | 5 | 3 | Yes | 1 | 5.2 |
|  | Least consistent | All | 1 | Yes | 1 | 17 |
| Cumulative mean | Most consistent | 5 | 3 | No | 1 | 1.9 |
|  | Average | 5 | 1 | Yes | 1 | 12 |
|  | Least consistent | 5 | 1 | No | 2 | 13 |
| Monthly mean | Most consistent | 5 | 2 | No | 1 | 5.8 |
|  | Average | 5 | 3 | No | 2 | 7.0 |
|  | Least consistent | All | 1 | No | 1 | 42 |
| Fixed threshold | Most consistent | - | 3 | - | 0.01 fixed | 0.0 |
|  | Average | - | 2 | - | 0.01 fixed | 0.0 |
|  | Least consistent | - | 1 | - | 0.01 fixed | 0.0 |

**Supplementary table S2. Parameter details of the most and least consistent definitions shown in Supplementary figure S4.** Parameters are explained in the methods section of the main manuscript. Fixed threshold methods use fixed values of incidence (100 or 300 cases per 10,000) instead of standard deviations above the mean.


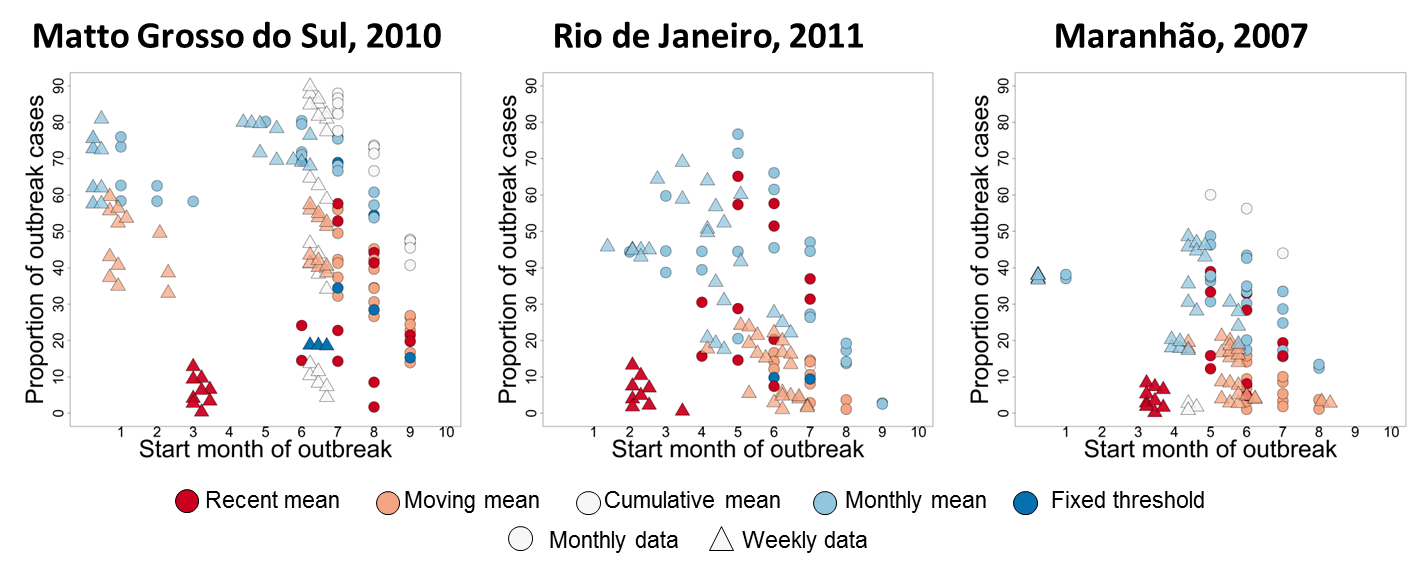


**Supplementary figure S5. The difference in time of onset (months since outbreak onset, x-axis) and overall outbreak size (y-axis) for three example outbreaks when all definitions are applied to weekly or monthly case data.** The results of definitions applied to monthly dengue case data are shown by circles, while those based on simulated weekly case data are shown as triangles. Different colour coding shows the division of these results by the different endemic channels. This figure is analogous to figure 5 in the main manuscript.

**Supplementary Results**

**Comparison of outbreak characteristics when standard outbreak definitions are applied to municipality instead of state level dengue case data**

The variation in outbreak characteristics is comparable whether standard outbreak definitions are applied to the 27 states of Brazil, or the 602 municipalities of São Paulo. The number of outbreaks identified in the two datasets is highly comparable with a slightly higher consistency when the definitions are applied to the municipality dataset (figure S1, upper panel). Despite this slightly higher consistency on the number of outbreaks, the proportion of cases that are identified as outbreak cases is considerably more variable when definitions are applied to the municipality dataset rather than the state-level dataset (figure S1, lower panel). Considering both measures, definitions applied at state level show greater consistency ($D=$ 11.8) than definitions applied at the municipality level ($D=$20.4).

Additionally, when just a single definition is applied across all states or municipalities the variation in the outbreak characteristics identified is greater amongst the municipality dataset than the state-level dataset (figure S2, $\hat{D}=$7.11 and $\hat{D}=$2.69 respectively). The difference between the most consistent, average and least consistent outbreak definition is also smaller when applied to states (figure S2, upper panel) as opposed to municipalities (figure S2, lower panel).

Both of these results could be exacerbated by the fact that there are a greater number of areas under examination in the municipality-level dataset (27 vs 602). This is, however, one practical component that needs to be considered when moving to a higher spatial scale, thus emphasising the increased problem of standardisation and comparability inherent in evaluating dengue outbreaks at this level.

**Comparison of outbreak characteristics when standard outbreak definitions are applied to weekly instead of monthly dengue case data**

When the whole range of standard definitions are applied to simulated weekly data, instead of reported monthly data, a similar proportion of cases are identified as outbreak cases (figure S3, lower panel), but the number of outbreaks differs (figure S3, upper panel). On average, outbreak definitions applied to weekly case data identify fewer outbreaks, however there are a smaller proportion of definitions that identify a very large number of outbreaks (figure S3, upper panel). This ensures that overall variability in outbreak number is higher when outbreak definitions are applied to weekly rather than monthly data ($\hat{D}=$48.0 and $\hat{D}=$11.8 respectively).

When just a single definition is applied to the weekly or monthly datasets from 27 different states, very similar levels of variation are seen (figure S4). Where a greater degree of consistency among a particular definition exists, it is usually found when definitions are applied to weekly data, but only identify a very small proportion of cases as an outbreak and are thus of questionable use as operationally relevant outbreak definitions (figure S4, lower panel, far right).

Finally, using weekly data does appear to lead to earlier detection of outbreak onset compared to using monthly data for the three outbreaks tested (figure S5). This difference is more pronounced in some outbreaks (Matto Grosso do Sul, Rio de Janeiro) than others (Marãnhao). This difference is also more pronounced for some endemic channel definitions, for example the recent mean method is improved in consistency and timing of response by using weekly rather than monthly data despite having a more limited use as an outbreak definition as it only detects a much smaller proportion of outbreak cases (figure S5). Despite these improvements in detection time and some specific examples of improved consistency, if all definitions are considered, using weekly as opposed to monthly datasets offers no improvement in outbreak definition consistency in terms of both proportion of outbreak cases or the month of outbreak onset.

**Supplementary Methods**

**Assembly of municipality level data for the state of São Paulo**

While the main focus of our analysis was to test the utility of standard outbreak definitions at wide-scale, state-level spatial resolutions, it is also likely that many dengue control programme coordinators at more local levels may also be using these standard outbreak definitions for decision making. Here we test the hypothesis that the amount of heterogeneity in outbreak characteristics identified by these standard definitions will only increase when applied to geographic units smaller than state-level. This hypothesis is based on the assumption that in areas with a lower population number, and therefore a lower probability of observing at least one case, the seasonal trend that the standard outbreak definitions attempt to capture will be more difficult to separate from stochastic variations in case infection and reporting and thus the characteristics of the outbreaks identified will be more inconsistent.

The state of São Paulo (B9) was chosen for the comparison between state-level and municipality level datasets due to its range of endemicity settings and its high population density [^1^](#_ENREF_1), which increases the chance of seasonal patterns being detectable over background stochastic cases. São Paulo is, therefore, likely to be one of the more suitable candidates for outbreak evaluation at the municipality-level. Monthly total dengue cases were extracted from January 2001 to December 2013 from Brazilian Ministry of Health surveillance system SINAN [^2^](#_ENREF_2)^,^[^3^](#_ENREF_3). A total of 602 municipalities reported at least one case of dengue over the time period. Missing data values were assigned a zero value. All definitions were applied to the new municipality-level data as previously stated in the Methods section of the main manuscript.

**Simulation of comparable weekly data from monthly datasets**

In a similar manner as above, the focus of our analysis was on wide-scale, state-level analyses where delays in data processing and assimilation may mean that dengue case data are unavailable at a higher temporal resolution than monthly records for evaluation in real-time. Under some circumstances, however, highly efficient electronic surveillance systems may have access to weekly data in real-time for decision making. This higher-resolution data may make elucidating seasonal trends more accurate, and in particular improve consistency among definitions about the start date of the onset of an outbreak. We hypothesise that, as above, the increased resolution will increase heterogeneity meaning that there is little or no improvement in consistency among standard outbreak definitions when using higher temporal resolution data.

Weekly reported dengue case data over comparable time periods and geographic areas is not publicly available, although it is available for selected weeks in 2013-2014 [^4^](#_ENREF_4). To characterise the variability in weekly case counts that make up the aggregated monthly values we evaluated the deviation of each weekly observation from the mean weekly value across a moving 4-week window period in the 2013-2014 weekly datasets. These deviations were then summarised by a normal distribution with mean 7.9 x 10^-3^ and standard deviation 4.5 x 10^-2^. This estimation method was then validated using bootstrap procedure where 70% of the data were used to predict against the holdout 30% of the data. The model gave good predictive performance with an $R^{2}$of the logged predictions of 0.88.

Monthly data from the main dataset was then converted to weekly values by sampling three values from this distribution scaled by the expected mean monthly values. A fourth weekly value was then computed based on the monthly value minus the sum of the previous three simulated values. Every fourth month contained five weeks ensuring an annual total of 52 weeks of observation. Standard outbreak definitions were applied to this weekly data using the same approach as with the monthly data, but with the week being the focus unit of analysis instead of the month.

**Supplementary Methods Bibliography**

1 Instituto Brasileiro de Geografia e Estatística. *IBGE: 2010 Population census results*, <<http://www.ibge.gov.br/english/estatistica/populacao/censo2010/default.shtm>> (

2 Ministério da Saúde. *Dengue: Notificações registradas no Sistema de Informação de Agravos de Notificação – SINAN, Brazil, 2001-2006*, <<http://dtr2004.saude.gov.br/sinanweb/tabnet/dh?sinan/dengue/bases/denguebr.def>> (

3 Ministério da Saúde. *Dengue: Notificações registradas no Sistema de Informação de Agravos de Notificação – SINAN, Brazil, 2007-2012*, <<http://dtr2004.saude.gov.br/sinanweb/tabnet/dh?sinannet/dengue/bases/denguebrnet.def>> (

4 Brasil Ministerio da Saude. *Boletim Epidemiológico - Volume 45*, <<http://portalsaude.saude.gov.br/index.php/situacao-epidemiologica-dados-dengue>> (2014).
